# Efficacy and safety of SHEN26, a novel oral small molecular RdRp inhibitor for COVID-19 treatment: a multicenter, randomized, double-blinded, placebo-controlled, phase II clinical trial

**DOI:** 10.1186/s12985-025-02631-y

**Published:** 2025-01-25

**Authors:** Jiangtao Bai, Tetsuya Asakawa, Wenfang Yuan, Yuanlong Lin, Hao Ju, Dandan Xu, Mingming Yang, Shuo Li, Guanguan Li, Deyin Guo, Hongzhou Lu, Xumu Zhang

**Affiliations:** 1 Shenzhen Kexing Pharmaceutical Co., Ltd, Shenzhen, Guangdong 518057 China; 2 Institute of Neurology, Shenzhen Third People’s Hospital, Second Hospital, Affiliated to Southern University of Science and Technology, Shenzhen, Guangdong 518112 China; 3 Department of Infectious Diseases, Shijiazhuang Fifth Hospital, Shijiazhuang, Hebei 050021 China; 4 Shenzhen Clinical Research Center for Infectious Disease, State Key Discipline of Infectious Disease, Shenzhen Third People’s Hospital, Second Hospital Affiliated to Southern University of Science and Technology, Shenzhen, 518112 China; 5 National Clinical Research Center for Infectious Disease, Shenzhen, 518112 China; 6 Department of Endocrinology, People’s Hospital of Longhua, Shenzhen, Guangdong 518109 China; 7 Medi-X Pingshan, Southern University of Science and Technology, Shenzhen, Guangdong 518118 China; 8 Shenzhen AntiV Pharma Co. Ltd., Shenzhen, Guangdong 518118 China; 9 Guangzhou National Laboratory, Guangzhou International BioIsland, Guangzhou, Guangdong 510320 China; 10 Department of Chemistry, Southern University of Science and Technology, Shenzhen, Guangdong 518055 China

**Keywords:** COVID-19, Efficacy and safety, Oral small molecular antiviral drug, RdRp inhibitor, Viral load

## Abstract

**Background:**

SHEN26 (ATV014) is an oral RNA-dependent RNA polymerase (RdRp) inhibitor with potential anti-SARS-CoV-2 activity. Safety, tolerability, and pharmacokinetic characteristics were verified in a Phase I study. This phase II study aimed to verify the efficacy and safety of SHEN26 in COVID-19 patients.

**Methods:**

This was a multicenter randomized double-blind placebo-controlled study. Mild-to-moderate adult patients with COVID-19 were recruited and randomly assigned to the high-dose (400 mg), low-dose (200 mg), or placebo groups (1:1:1). The primary outcome measure was “changes in RNA levels on Day seven (D7)”. The second outcome measures were “changes of RNA levels on D3, D5, D10, D28,” “Time of clearance of virus.”

**Results:**

A total of 91 patients were recruited in this study between December 08, 2022, and January 27, 2023. Twelve patients dropped out due to a lack of examination results. Finally, the data of 79 patients (24 in the placebo group, 31 in the 200 mg group, and 24 in the 400 mg group) were analyzed. No significant differences in the baseline data were observed between the groups. The changes of viral load were significantly higher on D3 (*P* = 0.0119), and D5 (*P* = 0.0120) in 400 mg group (vs. placebo group), and the difference value achieved 1.06 log10 copies/mL on D3 and 1.21 log10 copies/mL on D5. No significant difference was found in the viral clearance time between SHEN26 administrating groups and placebo groups. Administration of SHEN26 did not enhance drug-related ADEs and did not induce ADEs, and ADE inducing drug withdrawal, dose reduction, or death. Moreover, SHEN26 did not worsen the renal function.

**Conclusions:**

Our findings indicate a better efficacy of a high dose (400 mg) for COVID-19 treatment. These preliminary data on the efficacy and safety provide useful information and a working basis for further verification and development of SHEN26 as a novel oral small-molecule antiviral drug for treating COVID-19.

**Supplementary Information:**

The online version contains supplementary material available at 10.1186/s12985-025-02631-y.

## Introduction

It has been approaching five years since the COVID-19 pandemic, which has been a global public health concern. However, the threat and impact of COVID-19 cannot be ignored. According to the World Health Organization (WHO) dashboard, 776,205,140 infectors were reported worldwide in September 2024, and 65,018 infections increased in the previous seven days (on September 23, 2024) [1]. Many survivors suffer from sequelae of long COVID [2]. Hence, it is still an indispensable task to develop effective and safe agents to treat COVID-19, in which oral small-molecule drugs play a crucial role. Indeed, our clinical group was involved in several clinical trials verifying the efficacy and safety of novel oral small-molecule drugs in treating COVID-19, such as simnotrelvir, an oral 3-chymotrypsin-like protease inhibitor [3, 4].

RNA-dependent RNA polymerase (RdRp) plays a key role in viral replication and transcription. It has been considered as a target for the development of anti-COVID19 agents [5]. Of the agents targeting RdRp, remdesivir has been approved by the US Food and Drug Administration (FDA) for treating COVID-19 (approval date: May 01, 2020) [6]. Owing to the nature of intravenous administration, many researchers have considered the development of drugs targeting the parent nucleoside, namely 1′-CN-4-aza-7,9-dideazaadenosine C-nucleoside (GS-441524), against SARS-CoV-2 [5, 7, 8]. GS-441,524 reportedly has the effects suppresses viral titers in virus-infected organs without marked toxicity in SARS-CoV-2 infected mice [7]. Zhou et al. reported the anti- SARS-CoV-2 effects of SHEN26 (formerly known as ATV014), a potential oral nucleoside derived from GS-441,524 [8]. SHEN26 is orally administrated and forms GS-441,524, which has antiviral effects [9].

In a Phase I study of SHEN26 (NCT05504746, https://clinicaltrials.gov/study/NCT05504746, Registration Date: 2022-08-10), Sun et al. verified the safety, tolerability, and pharmacokinetic data of healthy Chinese participants [9]. They found that oral administration of SHEN26 (50–1200 mg once per day or 200–600 mg twice per day) for five consecutive days did not result in serious adverse events (ADEs, Common Terminology Criteria for Adverse Events [CTCAE] ≥ 2). No SHEN26-related hepatotoxicity was observed. After the oral administration of SHEN26, plasma SHEN26-69-0 rapidly reached its peak. After five days of SHEN26 administration, the concentration was stable without obvious accumulation [9]. Because the safety and tolerability of SHEN26 were proven to be satisfactory in the Phase I study [9], we designed and conducted a phase II study in China (NCT05676073, https://clinicaltrials.gov/study/NCT05676073, Registration Date: 2023-01-03) based on previous findings. We attempted to verify the efficacy and safety of SHEN26 in patients with mild-to-moderate COVID-19. Moreover, we attempted to determine the appropriate dose of SHEN26 for clinical application. According to the global design of the clinical trial of SHEN26, the present study did not compare the indices of symptom amelioration, which will be investigated in the following phase III study.

## Methods and materials

### Experimental design and patients

This multicenter, randomized, double-blind, placebo-controlled, phase II clinical trial was conducted in China. A total of 91 patients with COVID-19 from Dec 08 2022 to January 27, 2023, treated in several research centers, were recruited for this study. The inclusion criteria were as follows: *i*) adult patients (18 ≤ age ≤ 65 years) from inpatient, outpatient, and emergency department settings. In the present study, all recruited patients did not require additional oxygen therapy; *ii*) cycle threshold (CT) value of SARS-CoV-2-specific RT-PCR < 25 and the interval between the first PCR assay and administration of SHEN26 ≤ 5 days. *iii*) Patients with mild and moderate COVID-19 were determined according to the criteria of the Diagnosis and Treatment Plan for COVID-19 (version 9) issued by the National Health Commission of China [10]. We used the same definition as that in a previous study [3]. Briefly, patients with mild COVID-19 were defined as follows: (1) they must have the following conditions: respiration rate (RR) < 20/min, heart rate (HR) < 90/min, SPO_2_ > 93% on room air, or on non-COVID-19-related supplemental oxygen that did not increase since the onset of COVID-19. (2) They must not have shortness of breath at rest, exertion, respiratory failure, shock, or multi-organ dysfunction or failure. Patients with moderate COVID-19 were defined as follows: (1) they must have one or more of the following situations: *i)* shortness of breath or breath exertion, *ii)* RR ≥ 20 to < 30/min, and *iii)* HR ≥ to < 125/min. (2) They must have SPO_2_ > 93% on room air or on non-COVID-19-related supplemental oxygen, which has not increased since the onset of COVID-19. (3) Patients must not have shortness of breath at rest, breath exertion, respiratory failure, shock, or multi-organ dysfunction/failure. *iv*) indices of laboratory examination were hemoglobin ≥ 90 g/L, blood platelet count ≥ 100 × 10^9^, aspartic transaminase (AST) and alanine aminotransferase (ALT) ≤ 3 upper limit of normal (ULN), total bilirubin ≤ 2 ULN, and endogenous creatinine clearance rate > 60 mL/min. The exclusion criteria were: *i*) severe or critical COVID-19 patients; *ii*) patients undergoing mechanical ventilation; *iii*) patients whose SPO_2_ ≤ 93%, PaO_2_/FiO_2_ ≤ 300 mmHg, or breath rate ≥ 30/min, or rest heart rate ≥ 125/min; *iv*) reinfection patients who were infected with SARS-CoV-2 ≤ 3 months; *v*) those who underwent therapies against COVID-19 including monoclonal antibody therapy, antiviral therapy, plasma therapeutics, or human immunoglobulin treatment; *vi*) patients undergoing steroid drugs; *vii*) those who underwent dialysis; *viii*) those who underwent surgery of main organs within 28 days; *ix*) severe clinical conditions within 28 days in the other systems; *x*) those who accepted any vaccination within one month; *xi*) those who were recruited by the other clinical trials within one month; *xii*) other personal conditions such as refusal to sign the informed consent. This study was approved and supervised by the Ethical Committee of Shenzhen Third People’s Hospital (approval No: 2022-009), the Ethical Committee of Guangdong Second Provincial General Hospital (approval no: 2022-YWLCYJ-017-02), the Ethical Committee of Shijiazhuang Fifth Hospital (approval no: 2022-YW-004-1), and the Ethical Committee of People’s Hospital of Longhua Shenzhen (approval no: 2022-003Y-02PJ). All interventions were conducted in strict compliance with the protocol and the Declaration of Helsinki (2013). Signed informed consent was obtained from all participants before enrollment.

**Fig. 1:**
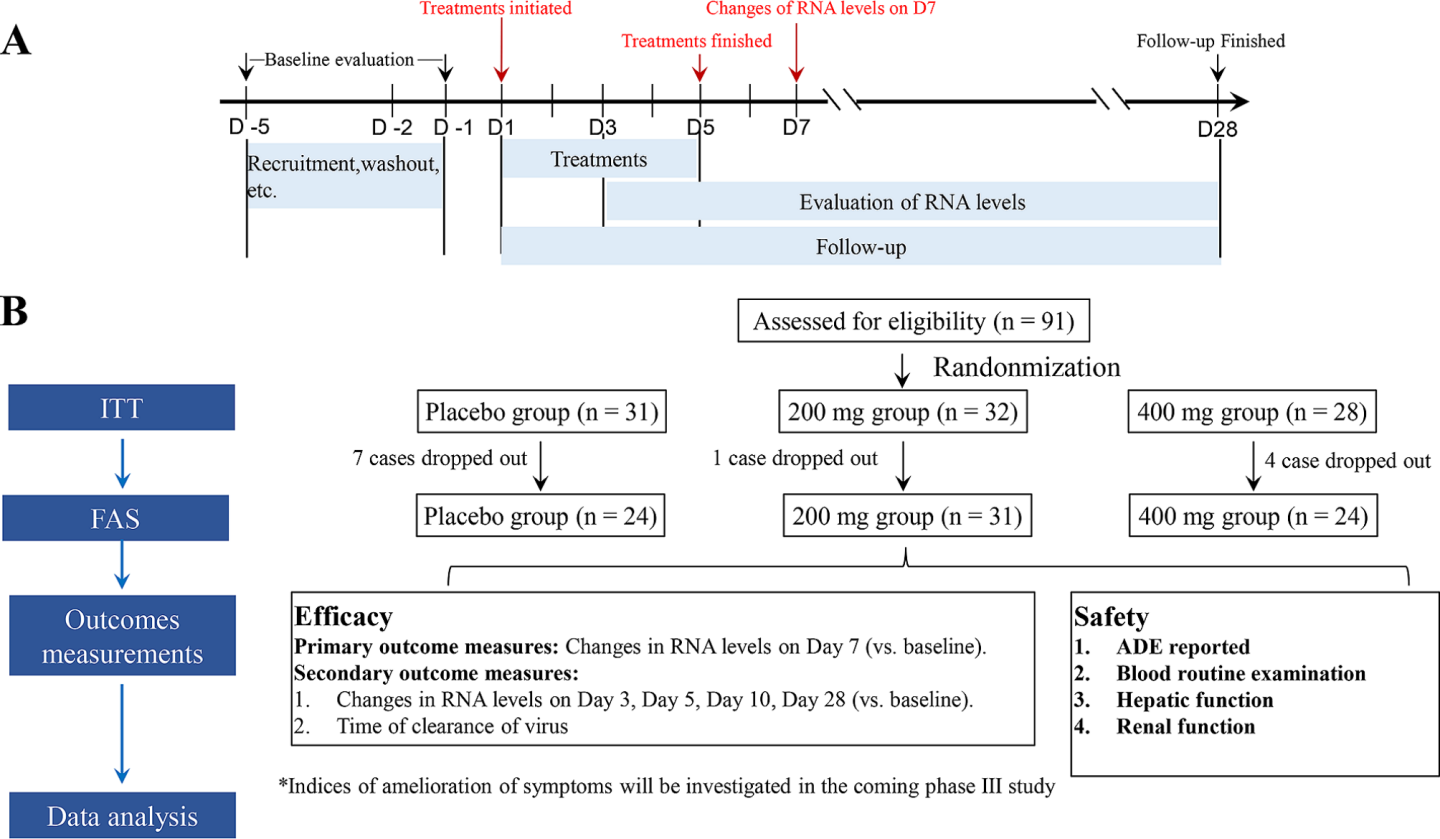
Protocol of the present study

The experimental design of this study is illustrated in Fig. 1. Randomization was performed using an interactive web response system (IWRS) to guarantee allocation concealment. Random numbers were generated using the PLAN procedure in SAS (version 9.4; SAS Institute, Cary, NC, USA) by an independent, unblinded statistician. Eligible patients were randomly allocated into three groups (1:1:1): placebo, 200 mg (low dose), and 400 mg (high dose) (Fig. 1). The dose of SHEN26 was selected according to the results of a Phase I study [9] and the clinical experience of physicians.

### Processes and outcome measurements

Baseline data, including the Demographic data demographic data, body weight, and body mass index (BMI), were collected once patients were enrolled in the study. The other data, including “viral load”, “COVID-19 severity, history of vaccination”, “time from administration to onset”, “high-risk factors for COVID-19”, “history of allergy”, “vaccination within 1 month”, “time since the last COVID-19 vaccination”, and “attention to other COVID-19 related clinical trials” were also collected. Indices of hepatorenal function included aspartate aminotransferase (AST), alanine aminotransferase (ALT), blood urea nitrogen (BUN), serum uric acid (UA), and serum creatinine (Cr). Drug administration was initiated on day one (D1) and sustained for five days (D1–D5). Drugs were orally administrated twice a day after dinner after meals, and the interval between administrations was 12 h [9]. In terms of efficacy, the primary outcome measures were the changes in RNA levels on D7, while the secondary outcome measures were: *i*) changes in RNA levels on D3, D5, D10, and D28 (if available), and *ii*) time of virus clearance. In terms of safety, all treatment-emergent adverse events (TEAEs) were recorded from D1. The severity of ADEs was classified into five classes according to the CTCAE (version 5.0) [11]. Drug-related adverse drug events (ADEs) were determined by a blinded clinician. Moreover, indices of hepatorenal function after treatment (D5 and D7) were measured to evaluate the potential effects of the drugs on hepatorenal function (Fig. 1).

### Statistics

Statistical analyses were performed using the SAS system SAS (version 9.4, SAS Institute, Cary, NC, USA). The normal distribution of continuous variables was confirmed using the Shapiro-Wilk test. Data are presented as mean ± standard deviation (SD). The Student’s t-test was used to compare data between the groups. Chi-square test or Fisher’s precision probability test was used to compare the rates between the two groups. The changes in SARS-CoV-2 viral RNA levels from baseline at each visiting point were analyzed using the analysis of covariance (ANCOVA) method. The Kaplan-Meier method was used to analyze the time to clearance of SARS-CoV-2 virus. Hazard ratios (HR) of the time-to-event endpoints were calculated using a proportional hazards (PH) Cox regression model. Statistical significance was set at *P* < 0.05.

## Results

### Clinical characteristics and baseline data

A total of 91 patients were recruited in this study; of these, 12 were excluded due to a lack of examination results at the endpoint of therapy. Finally, 79 patients (24 in the placebo group, 31 in the 200 mg group, and 24 in the 400 mg group) were analyzed (Fig. 1B). The clinical characteristics and baseline data of the enrolled patients are presented in Table 1. All patients had mild (76 of 79, 96.2%) and moderate (3 of 79, 3.80%) disease, and none of the patients with severe disease were included in this study. General information, such as sex distribution, body weight, BMI, and viral load, did not exhibit significant differences among the three groups. With respect to domains such as severity of COVID-19, history of vaccination, time from administration to onset, high-risk factors of COVID-19, history of allergy, vaccination within 1 month, time since the last COVID-19 vaccination, and attention of other COVID-19-related clinical trials, no significant differences were found among the three groups (Table 1). These results indicated satisfactory homogeneity among the three groups.

**Table 1:** Clinical characteristics and baseline data of the participants

Variables	Placebo group (*n* = 24)	200 mg group (*n* = 30)	400 mg group (*n* = 24)	P1	P2	P3
**General information**						
Gender (%)						
Male	21 (87.5)	19 (61.3)	16 (66.7)	0.0304	0.0860	0.6810
Female	3 (12.5)	12 (38.7)	8 (33.3)			
Age (years)	30.4 ± 7.61	35.9 ± 12.50	33.5 ± 10.50	0.0653	0.2502	0.4585
Body weight (kg)	67.75 ± 11.79	67.22 ± 12.97	62.03 ± 10.34	0.8780	0.0807	0.1145
BMI (kg/m^2^)	23.39 ± 3.09	23.76 ± 3.81	22.18 ± 2.68	0.6999	0.1535	0.0895
Viral load (log_10_ copies/mL)	7.13 ± 1.02	6.62 ± 1.52	7.41 ± 0.85	0.1665	0.2964	0.0259
**COVID-19 severity** (%)						
Mild	23 (95.8)	31 (100.0)	22 (91.7)	0.2514	0.5510	0.1016
Moderate	1 (4.2)	0	2 (8.3)			
Severe	0	0	0			
**History of vaccination** (%)						
Yes	23 (95.8)	29 (93.5)	22 (91.7)	0.7113	0.5510	0.7898
No	1 (4.2)	2 (6.5)	2 (8.3)			
**Time from administration to onset (days)**	4.8 ± 0.79	4.5 ± 0.81	5.0 ± 1.38	0.2894	0.4464	0.1106
**High risk factors of COVID-19** (%)				0.3745	NA	0.3745
Yes	0	1 (3.2)	0			
No	24 (100.0)	30 (96.8)	24 (100.0)			
**History of allergy** (%)						
Yes	2 (8.3)	0	0	0.1016	0.1486	NA
No	22 (91.7)	31 (100.0)	24 (100.0)			
**Vaccination within 1 month** (%)						
Yes	0	0	0	NA	NA	NA
No	24 (100.0)	31 (100.0)	24 (100.0)			
**Time since the last COVID-19 vaccination (days)**	23	29	22	0.1007	0.3704	0.4946
**Attention of the other COVID-19 related clinical trials** (%)				0.2514	> 0.9999	0.2514
Yes	1 (4.2)	0	1 (4.2)			
No	23 (95.8)	31 (100.0)	23 (95.8)			

*P1* = 200 mg group vs. Placebo, *P2* = 400 mg group vs. Placebo, *P3* = 200 mg group vs. 400 mg group

### Efficacy of SHEN26

**Fig. 2:**
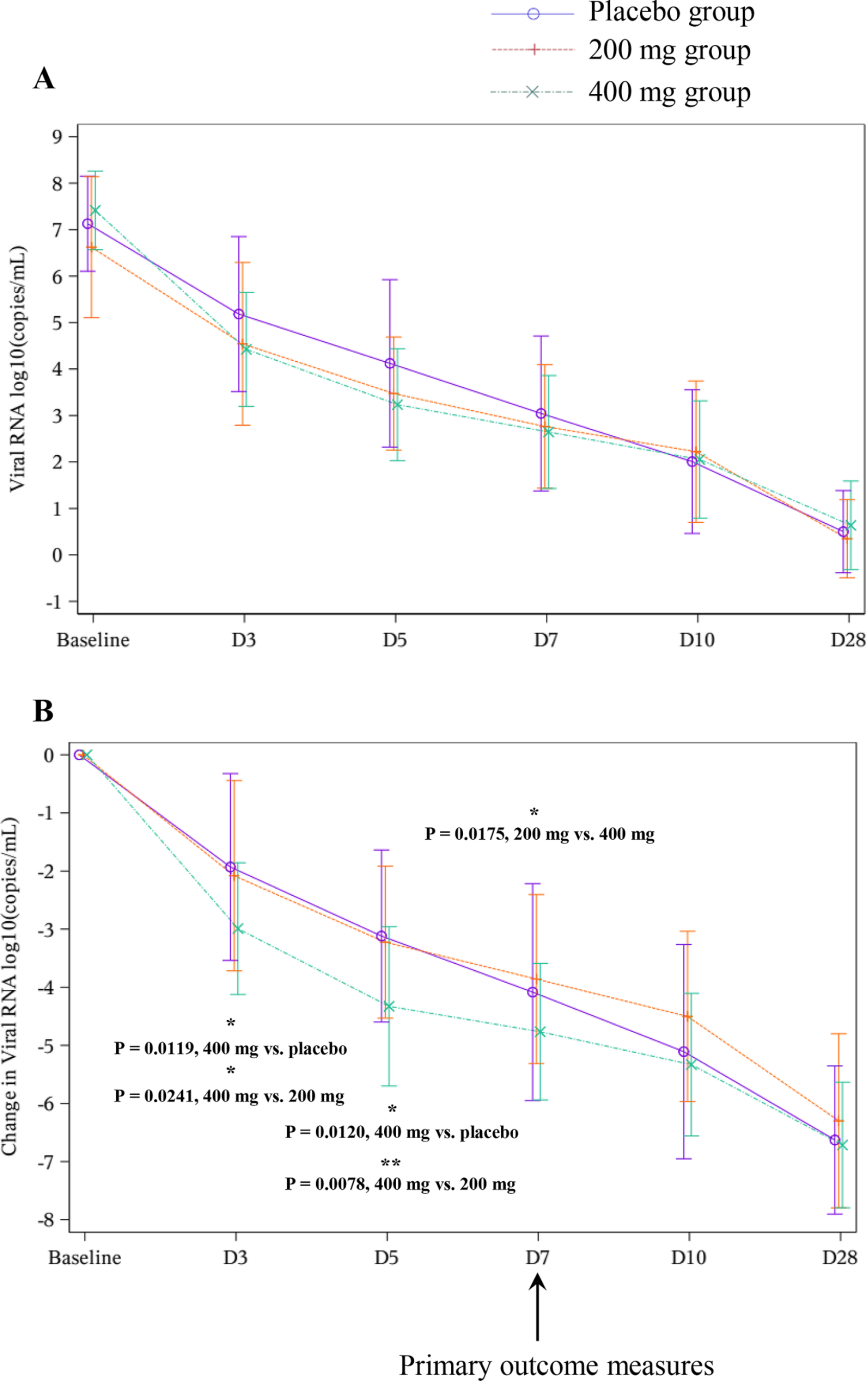
Dynamic changes of RNA levels after administration of SHEN26. **A**. Dynamic changes in RNA levels on different days after administration of SHEN26. **B**. Dynamic changes of difference in RNA levels (Days-baseline) after administration of SHEN26

In terms of the primary outcome measures, changes in RNA levels after administration of SHEN26 on D7 were significantly different between the 200 mg and 400 mg groups; 400 mg of SHEN26 achieved better effects in reducing the viral load, although both groups did not exhibit significant reduction compared with the placebo group. In addition, on D3, 400 mg group achieved significant reduction compared with placebo group and 200 mg group (-2.99 ± 1.13 vs. -1.93 ± 1.61, *P* = 0.0119; -2.99 ± 1.13 vs. -2.08 ± 1.64, *P* = 0.0241 respectively). On D5, 400 mg group achieved significant reduction compared with placebo group and 200 mg group (-4.33 ± 1.37 vs. -3.12 ± 1.48, *P* = 0.0120; -4.33 ± 1.37 vs. -3.22 ± 1.31, *P* = 0.0078 respectively). The difference value achieved 1.06 log10 copies/mL on D3 and 1.21 log10 copies/mL on D5. These results demonstrated that the administration of 400 mg SHEN26 efficiently reduced the viral load at an early stage (D3–D5). The effects of SHEN 26 were dose dependent (Fig. 2 and Table S1).

**Fig. 3:**
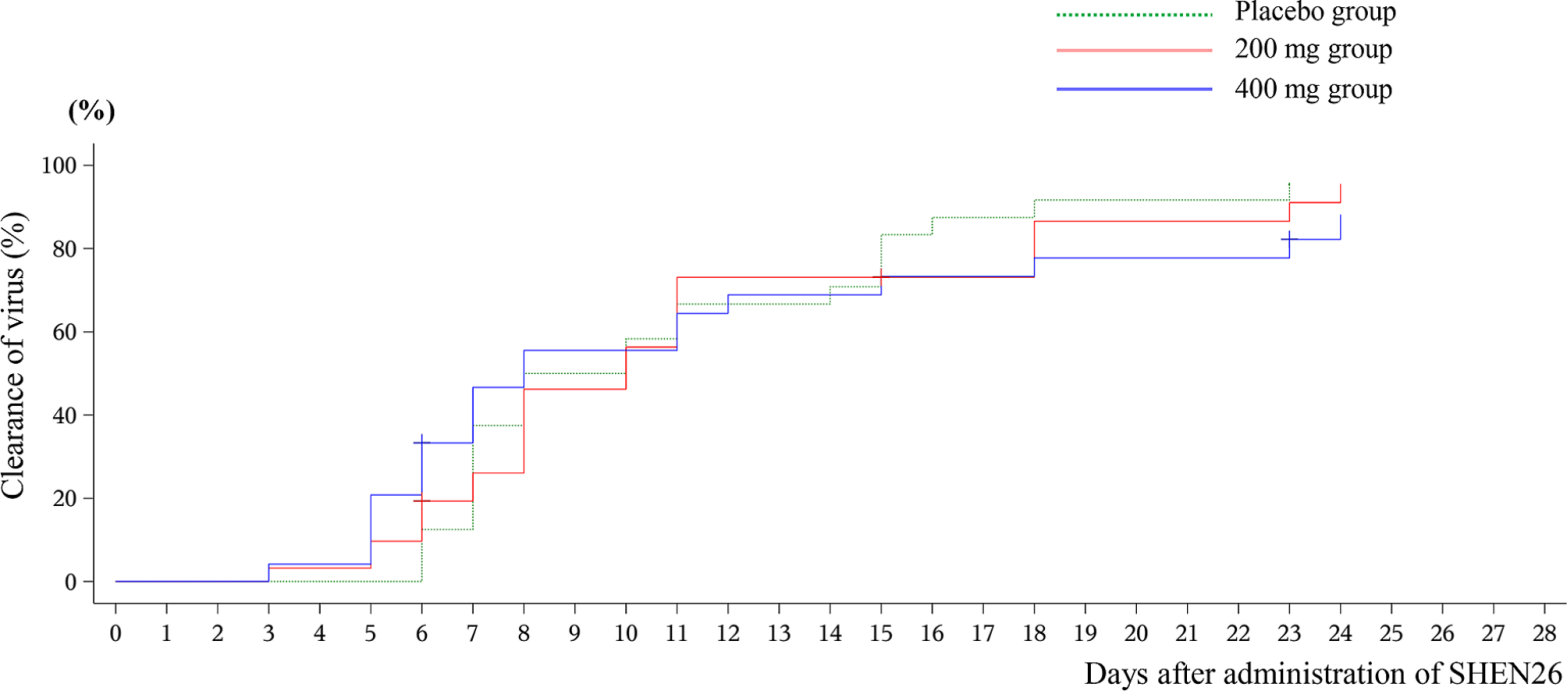
Times of Clearance of virus after administration of SHEN26

In terms of the indices of time of virus clearance, no significant difference was found between the 200 mg (*P* = 0.1275, 0.2160, 0.7808, and > 0.9999 on D3, D5, D7, and D10, respectively) and 400 mg and placebo group (*P* = 0.1092, 0.1818, 0.2443, and 0.7557 on D3, D5, D7, and D10, respectively). However, the 400 mg group exhibited a trend of a higher clearance rate within ten days after administration (Fig. 3).

### Safety of SHEN26

As shown in Table 2, the total number of TEAEs reported in the placebo, 200 mg, and 400 mg groups was 45, 51, and 29, respectively. Of these, the total cases of CTCAE ≥ 3 in the placebo, 200 mg, and 400 mg groups were 1, 0, and 3, respectively. Only cases of severity 1 in the 200 mg group were significantly higher than those in the placebo group (*P* = 0.0107). Drug-related ADEs in the placebo group, 200 mg group and 400 mg group were 13, 21, and 14, respectively. All SHEN26 drug-related ADEs were of grade < 3. No significant difference was found among the placebo, 200 mg, and 400 mg groups. No ADE or ADE-inducing drug withdrawal, ADE dose reduction, or ADE-causing deaths have been reported. (Table 2).

**Table 2:** Reported treatment emergent adverse events (TEAEs)

Variables	Placebo group (*n* = 24)	200 mg group (*n* = 31)	400 mg group (*n* = 24)	P1	P2	P3
Total TEAEs reported	45	51	29			
Severity (%) of TEAEs						
1	5 (20.8)	17 (54.8)	10 (41.7)	0.0107*	0.1195	0.3325
2	7 (29.2)	4 (12.9)	2 (8.3)	0.1804	0.1365	0.6862
3	0	0	2 (8.3)	NA	0.4894	0.1859
4	1 (4.2)	0	1 (4.2)	0.4364	> 0.9999	0.4364
5	0	0	0	NA	NA	NA
Total cases of CTCAE ≥ 3	1 (4.2)	0	3 (12.5)	0.4364	0.6085	0.0771
Drug related ADEs	13 (54.2)	21 (67.7)	14 (58.3)	0.3041	0.7711	0.4719
Several ADE	0	0	0	NA	NA	NA
ADE inducing drug withdrawal	0	0	0	NA	NA	NA
ADE inducing reduction of dose	0	0	0	NA	NA	NA
ADE causing death	0	0	0	NA	NA	NA

ADE, adverse event; CTCAE, Common Terminology Criteria for Adverse Events; NA, not available; TEAE, treatment-emergent adverse event

*P1* = 200 mg group vs. Placebo, *P2* = 400 mg group vs. Placebo, *P3* = 200 mg group vs. 400 mg group. * means *P* < 0.05

We also assessed the hepatorenal function before and after treatment. With regard to hepatic function, on D5 (just finished treatment), we did not find that hepatic function was deteriorated by SHEN26. The only significant difference was found in the AST of placebo group, the placebo treatment significant lowered the AST levels (after 24.51 ± 6.11 U/L vs. before 30.33 ± 12.83 U/L, *P* = 0.0340). As far as the renal function, we did not find the renal function was worsened by the SHEN26. The significant differences were found in the Cr of placebo group and 400 mg group, but the data of Cr after treatment were even lower (after 74.55 ± 13.91 µmol/L vs. before 82.43 ± 15.73 µmol/L, *P* = 0.0438 in placebo group, and after 69.83 ± 12.86 µmol/L vs. before 74.00 ± 14.04 µmol/L, *P* = 0.0112 in 400 mg group). With respect to the data on D7 (two days after treatment), no significant difference was found between the 200 mg and 400 mg groups. The only significant difference was observed in the placebo group. The UA levels were higher by the placebo (after 389.94 ± 72.92 µmol/L vs. before 377.45 ± 108.97, *P* = 0.0123).

## Discussion

Based on previous findings of SHEN26 in a preclinical study [8] and Phase I study [9], this multicenter, randomized, double-blinded, placebo-controlled, Phase II clinical trial (NCT05676073) verified the efficacy and safety of SHEN26 in mild-to-moderate Chinese patients with COVID-19. In addition, we attempted to determine the appropriate dose of SHEN26 in a clinical setting. First, we confirmed the efficacy of the SHEN26. We found that at the early stage of administration (on D3 and D5),400 mg group had better efficacy in lowering the viral load than the placebo and 200 mg groups. Second, we confirmed the safety of the SHEN26. No significant difference in the total number of cases of severe TEAEs (CTCAE ≥ 3) and drug-related ADEs was found among the placebo, 200 mg, and 400 mg groups. No ADE-related withdrawals, dose reductions, deaths, or severe ADEs were reported. Importantly, our results confirmed that neither dose of SHEN26 worsened the hepatorenal function. Thus, our results suggest that 400 mg SHEN26 may be an appropriate dose for clinical use. To the best of our knowledge, this is the first clinical trial on SHEN26. Despite there are several limitations, we believe that the findings of this study provide useful information for further verification of SHEN26 as a novel anti-SARS-CoV-2 drug.

### Efficacy

The main positive finding of the present study is the large dose of SHEN26 (400 mg per day, 200 mg b.i.d.), which reduced the viral load at the early stage of administration (on D3 and D5, Fig. 2 and Table S1). The other outcome measures including “time of clearance time” did not exhibited significant difference (Fig. 3). These results are in agreement with analogous studies of other oral small-molecule antiviral drugs such as simnotrelvir [3] and SIM0417 [4]. These oral small-molecule antiviral drugs seem to have the same effects, namely, a reduction of the viral load at the early stage of administration (≤ 5 days). This suggests that SHEN26 may be useful against SARS-CoV-2 by decreasing viral load at an early stage. Owing to the strict nature of the COVID-19 control policy in China at that time, most patients could be identified and treated at a very early stage. The time from drug administration to disease onset was approximately 5 days (Table 1). Our results verified the value of SHEN26 in the early stages of COVID-19 infection. However, our data did not identify any benefits regarding the viral clearance time. One plausible interpretation is the change in anti-epidemic measures at that time. Indeed, it was difficult to determine the precise timing of viral clearance because RT-PCR frequency was reduced. Another plausible interpretation is the small sample size of the present study. Many factors are considered to affect the clinical manifestations of COVID-19, and small sample sizes may lack representativeness and lead to biased results. Indeed, in terms of the problem of “whether the antiviral medications are helpful in shortening the disease course,” the answers in previous trials are heterogeneous. For example, serial reports from Pfizer Inc. claimed that nirmatrelvir plus ritonavir did not accelerate the amelioration of 11 symptoms [12, 13]. However, other studies have concluded that oral administration of small-molecule drugs reduces the duration of symptoms [3, 4, 14, 15]. To clarify these issues, a multicenter study with a large sample size is required. However, the present study was conducted between Dec 08 2022–and January 27, 2023, when the epidemic prevention policy against COVID-19 in China was changing. It was difficult to recruit a large number of hospitalized patients who could be rigorously managed, treated, and observed according to the protocol in the clinical setting. In this regard, the present study recruited patients including inpatient, outpatient, and emergency department settings. Accordingly, the present study could not obtain robust evidence regarding whether SHEN26 can reduce the disease course, which warrants further investigation. Moreover, whether SHEN26 is beneficial in ameliorating COVID-related symptoms will be investigated in a future phase III study.

### Safety

Based on our Phase I study demonstrating the satisfactory safety of SHEN26 in healthy subjects [9], this study verified the safety of SHEN26 in patients with mild to moderate COVID-19. In terms of TEAEs, the only significant difference was found in CTCAE great 1 between the 200 mg and placebo groups. All SHEN26-related drug related ADEs were grade < 3. No significant difference in drug-related ADEs was found among the placebo, 200 mg, and 400 mg groups. In addition, no ADEs or ADE-induced drug withdrawal, dose reduction, or death were reported (Table 2). These results are in accordance with those of previous studies on oral small-molecule antiviral drugs [3, 4]. Moreover, this study did not find that the administration of SHEN26 deteriorated hepatorenal function. The most significant differences were observed in the placebo group. The only difference in the SHEN26 groups was the Cr level in the 400 mg group on D5, but the level of Cr was lower than that before administration (Table 3). Thus, the safety of SHEN26 was confirmed. Along with previous studies [3, 4], this study demonstrated the satisfactory safety of oral small-molecule antiviral drugs.

**Table 3:** Changes of hepatorenal functions before and after treatments

Groups	Date after treatment	Hepatorenal functions				
		Hepatic function		Renal function		
		AST (U/L)	ALT(U/L)	BUN (mmol/L)	UA (mmol/L)	Cr
Placebo group	Before	30.33 ± 12.83	34.83 ± 19.05	4.870 ± NA	377.45 ± 108.97	82.43 ± 15.73
	D5	24.51 ± 6.11	29.71 ± 10.79	NA	391.09 ± 93.750	74.55 ± 13.91
	*P*(D5 vs. Before)	0.0340*	0.2354	NA	0.9226	0.0438*
	D7	22.16 ± 8.51	30.66 ± 23.35	5.01 ± NA	389.94 ± 72.92	79.25 ± 13.54
	*P*(D7 vs. Before)	0.0532	0.3547	NA	0.0123*	0.0767
200 mg group	Before	25.19 ± 10.33	25.66 ± 17.58	3.35 ± 0.45	381.04 ± 112.30	73.30 ± 15.06
	D5	24.76 ± 13.99	28.07 ± 20.25	4.38 ± 0.75	390.07 ± 87.58	69.21 ± 12.64
	*P*(D5 vs. Before)	0.9082	0.6062	0.2664	0.9131	0.1378
	D7	22.53 ± 8.02	24.14 ± 13.97	4.35 ± 0.31	378.32 ± 100.80	71.66 ± 16.07
	*P*(D7 vs. Before)	0.9436	0.5377	0.1402	0.4006	0.7268
400 mg group	Before	27.80 ± 9.10	27.25 ± 17.71	4.300 ± 0.54	344.51 ± 79.62	74.00 ± 14.04
	D5	24.63 ± 6.74	25.53 ± 10.64	4.925 ± 0.77	367.49 ± 75.89	69.83 ± 12.86
	*P*(D5 vs. Before)	0.1415	0.7478	0.6217	0.2814	0.0112*
	D7	26.99 ± 6.86	33.24 ± 19.93	4.36 ± 0.30	422.99 ± 79.75	75.73 ± 16.57
	*P*(D7 vs. Before)	0.7706	0.6828	0.9355	0.0538	0.1741

ALT, alanine aminotransferase; AST, aspartate transaminase; BUN, blood urea nitrogen; Cr, creatinine; NA, not available; UA, uric acid

* means *P* < 0.05

### Strengths and limitations

This study has several strengths. This is the first multicenter, randomized, double-blind, placebo-controlled, clinical trial to verify the efficacy and safety of SHEN26, a novel RdRp-targeted oral small-molecule antiviral drug. Our results showed that a high dose of 400 mg might be a better dose for clinical use (vs. a low dose of 200 mg). In addition, our results preliminarily verified the value of SHEN26 in the treatment of COVID-19 at an early stage.

Nevertheless, this study has several limitations. First, the small sample size (only 79 cases) might be a contributing factor to obtaining robust evidence. In particular, for indices that might be influenced by many factors, a small sample size might induce a biased conclusion. Second, most participants had mild disease, and only three moderate cases were enrolled in this study. The unbalanced distribution of patients might have selection bias, although most of the patients were mild, as the mainstream strain during the research period was Omicron BA. Despite these limitations, the results of this study are valuable because they provide a working basis for further verification of the efficacy and safety of SHEN26 as an emerging oral small-molecule antiviral drug against COVID-19.

## Conclusions

In the present phase II study, we confirmed the preliminary efficacy and safety of SHEN26 for treating patients with mild-to-moderate COVID-19 in China. We demonstrated that administration of SHEN26 at a high dose (400 mg) significantly reduced the viral load at the early stage of administration (on D3 and D5). However, this viral benefit was not confirmed at the following time points. Administration of SHEN26 did not enhance drug-related ADEs and did not induce severe ADEs, and ADE inducing drug withdrawal, dose reduction, or death. Moreover, the administration of SHEN26 did not worsen hepatorenal function. These preliminary data on the efficacy and safety provide useful information and a working basis for further verification and development of SHEN26 as a novel oral small-molecule antiviral drug for treating COVID-19.

## Electronic supplementary material

Below is the link to the electronic supplementary material.

Supplementary Material 1

## Data Availability

The datasets used and/or analyzed during the current study are available from the corresponding author upon reasonable request.

## Declarations

### Clinical trial registration

ClinicalTrials.gov ID: NCT05676073, https://clinicaltrials.gov/study/NCT05676073, Registration Date: 2023-01-03.
