# Supplementary material for: Efficacy and safety of SHEN26, a novel oral small molecular RdRp inhibitor for COVID-19 treatment: a multicenter, randomized, double-blinded, placebo-controlled, phase II clinical trial
Source: Virol J. 2025 Jan 25;22:16. doi: 10.1186/s12985-025-02631-y (PMC11762885; doi:10.1186/s12985-025-02631-y)
Supplement: Supplementary file 1 — Supplementary Material 1 [file 12985_2025_2631_MOESM1_ESM.docx]

**Supplementary Materials**

**Table S1.** Changes of SARS-CoV-2 RNA levels (Day n- Baseline) on day 3, day 5, day 7, day 10, day 28 (Day n - Baseline)

| **Days** | **ΔRNA Levels (log 10 copies/mL)** | | | | | |
| --- | --- | --- | --- | --- | --- | --- |
|  | **Placebo group (n=24)** | **200 mg group (n=31)** | **400 mg group (n=24)** | ***P1*** | ***P2*** | ***P3*** |
| **3** | -1.93 ± 1.61 | -2.08 ± 1.64 | -2.99 ± 1.13 | 0.7382 | 0.0119* | 0.0241* |
| **5** | -3.12 ± 1.48 | -3.22 ± 1.31 | -4.33 ± 1.37 | 0.7949 | 0.0120* | 0.0078** |
| **7** | -4.08 ± 1.87 | -3.86 ± 1.45 | -4.77 ± 1.17 | 0.6173 | 0.1432 | 0.0175* |
| **10** | -5.11 ± 1.84 | -4.50 ± 1.47 | -5.33 ± 1.23 | 0.2210 | 0.6574 | 0.0529 |
| **28** | -6.63 ± 1.28 | -6.30 ± 1.50 | -6.71 ± 1.08 | 0.4080 | 0.8058 | 0.2832 |

*P1* = 200 mg group vs. Placebo, *P2* = 400 mg group vs. Placebo, *P3* = 200 mg group vs. 400 mg group, * *P* < 0.05, ** *P* < 0.01
